# Patient and health care system characteristics are associated with delayed treatment of tuberculosis cases in Taiwan

**DOI:** 10.1186/s12913-019-4702-0

**Published:** 2019-11-19

**Authors:** Chien-Chou Chen, Po-Huang Chiang, Yen-Hsu Chen, I-Chun Fan, Ta-Chien Chan

**Affiliations:** 1 Center for Applied Artificial Intelligence Research, Soochow University, Taipei, Taiwan; 2 Institute of Population Health Sciences, National Health Research Institutes, Zhunan, Taiwan; 3 Department of Internal Medicine, Kaohsiung Municipal Ta-Tung Hospital, Kaohsiung, Taiwan; 4 School of Medicine, Graduate Institute of Medicine, Sepsis Research Center, Center of Tropical Medicine and Infectious Diseases, Kaohsiung Medical University, Kaohsiung, Taiwan; 5 Department of Biological Science and Technology, College of Biological Science and Technology, National Chiao Tung University, Hsin Chu, Taiwan; 6 Institute of History and Philology, Academia Sinica, Taipei, Taiwan; 7 Research Center for Humanities and Social Sciences, Academia Sinica, Taipei, Taiwan; 8 Institute of Public Health, School of Medicine, National Yang-Ming University, Taipei, Taiwan; 9 Division of Infectious Diseases, Department of Internal Medicine, Kaohsiung Medical University Hospital, Kaohsiung, Taiwan

**Keywords:** Tuberculosis, Patient-pathway analysis, Health system delay

## Abstract

**Background:**

The decline of the incidence rate of tuberculosis in Taiwan has been partly attributed to the launch of the directly observed therapy short course (DOTS) program in 2006, followed by the DOTS-Plus in 2007. However, with the phasing out of the specialized tuberculosis care system and the declining incidence, clinical workers in Taiwan might become less familiar with the presentation of tuberculosis. Complementing the patient-pathway analysis with health system delay estimates, the objective of this study is twofold: to estimate the alignment between patient care initiation and the availability of prompt diagnostic and treatment services, and to identify the risk factors of delayed tuberculosis treatment.

**Methods:**

The study population included all Taiwanese patients with incident tuberculosis in 2013. We (1) identified 11,507 incident tuberculosis patients from the 2013 National TB Registry, and (2) linked 10,932 Taiwanese from the registry to the 2012–2013 National Health Insurance Research Database. We assessed patient’s care-seeking pathways and associated the determinants of health system delay in a Cox model.

**Results:**

The overall health system delay was 46 days. We found that 20.5 and 3.5% of 10,932 tuberculosis patients were diagnosed and treated respectively at the initial visit to seek care for TB-related symptoms. Risk factors related to the prolonged health system delay included female gender (adjusted HR = 0.921, 95% CI: 0.884, 0.960), age > =65 years (adjusted HR = 0.720, 95% CI: 0.692, 0.750), non-severe (chest X-ray without cavities) (adjusted HR =0.721, 95% CI 0.683–0.760), chronic respiratory diseases (adjusted HR = 0.544, 95% CI: 0.522, 0.566), living in long-term care facilities (adjusted HR = 0.580, 95% CI: 0.525,0.640), an initial visit at a primary care clinic (adjusted HR = 0.588, 95% CI: 0.565, 0.612), and living in southern Taiwan (adjusted HR = 0.887, 95% CI: 0.798, 0.987).

**Conclusions:**

The low access to TB diagnostic and treatment services at the initial visit and the prolonged health system delay indicate inefficiency in the health care system. Strengthening training of physicians at public hospitals and health workers at nursing homes might improve the efficiency and timeliness of tuberculosis diagnosis and treatment in Taiwan.

## Background

**Fig. 1:**
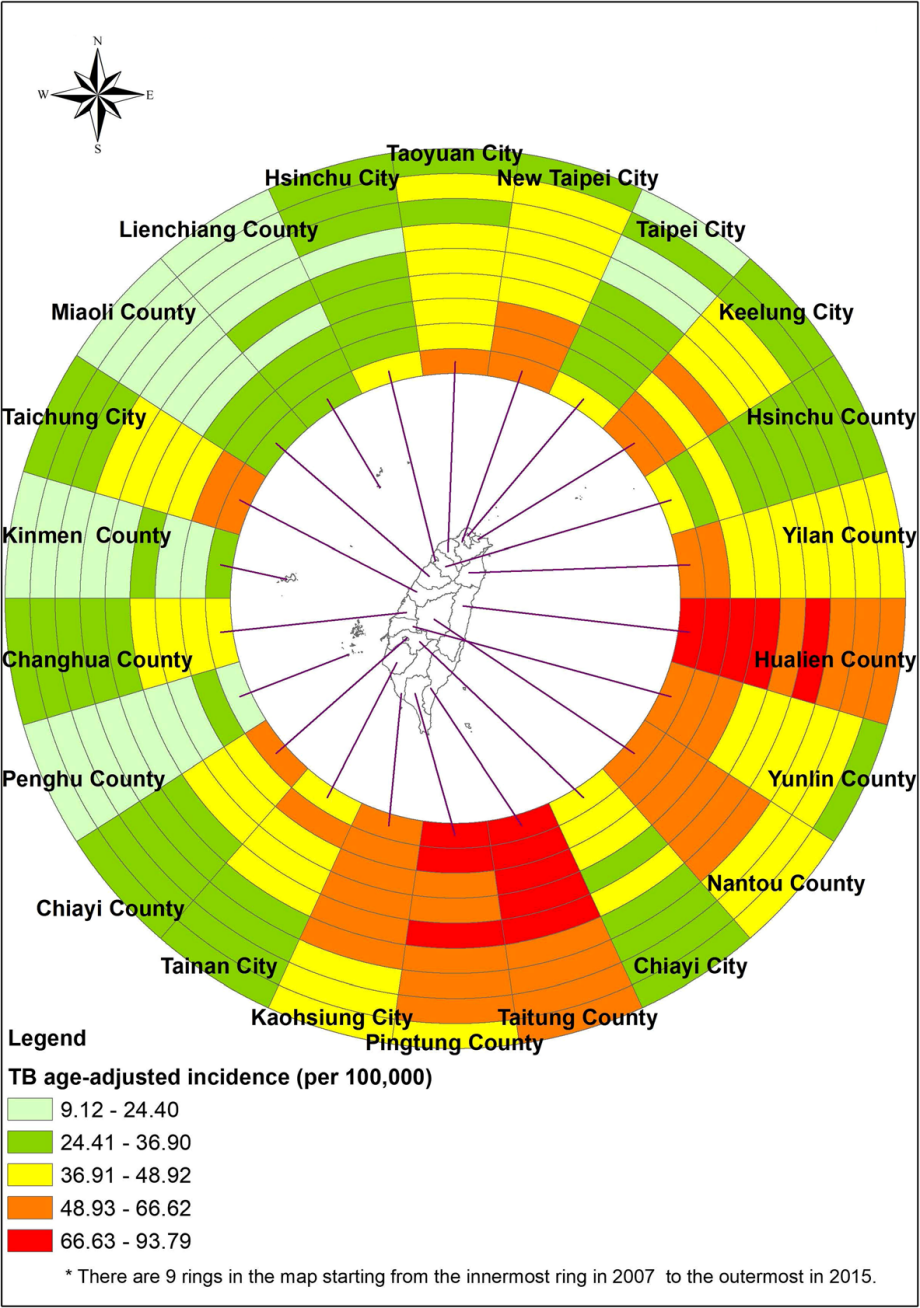
Tuberculosis incidence rates in Taiwan. The map presents tuberculosis incidence in a compelling manner by year (2007–2015 from inner to outer rings) and region (*n* = 22)

Since the health sector reform in 2002, Taiwan has integrated the previous vertical tuberculosis (TB) control system into the general health care system [1]. The incidence rate of TB has gradually decreased from 63.2 (per 100,000 people) in 2007 to 45.7 in 2015 [2]. The declining incidence rate has been partly attributed to the launch of the directly observed therapy short course (DOTS) program in 2006, followed by the DOTS-Plus for multidrug-resistant TB in 2007, preventing the spread of TB bacilli [3, 4]. Recently, a new regimen of 3 months of isonianizid plus rifapentine for latent TB infection (LTBI) has been introduced into Taiwan, contributing to the ongoing eradication of TB [5]. However, TB incidence in 2007–2015, portrayed by a ring map (Fig. 1), was heterogeneous at the city and county levels.

With the phasing out of the specialized TB care system and the declining TB incidence, it is likely that more clinical workers will become less familiar with the presentation of TB [1]. Meanwhile, if the general health care system is not efficient in promptly identifying patients with TB, the patients might need to make repeated visits within the health care system for a prolonged period before being diagnosed with TB [6]. It is even worse that frequent health care visits of nontuberculous patients appear to be a risk factor for contracting TB [7, 8].

The objective of the study is twofold: to estimate the gap between care initiation and availability of TB services, and to identify the risk factors of delayed TB treatment. A patient-pathway analysis (PPA) assesses the alignment between patient care initiation and the availability of prompt TB diagnostic and treatment services [9–13]. In addition, health system delay [14, 15] (HSD) analysis measures the delay between the initial visit to seek care for TB-related symptoms and the initiation of TB treatment. Complementing PPA with HSD estimates, our assessment addresses the amount of access to TB services and further unveils the determinants of delay. Furthermore, the sector effect (public against private) and health facility type (hospital against primary care clinic) on TB services are also revealed [16]. The results might guide prioritization of regions or hospitals for intensified engagement in the general health care system.

## Methods

### Ethics

This study was approved by the institutional review board (IRB) of Kaohsiung Medical University Chung-Ho Memorial Hospital for research ethics (IRB#: KMUHIRB-SV (I)-20,160,057). The data used in this study were derived from linkage of two databases including the National Health Insurance Research Database (NHIRD) and the TB registry. Trained staff from the Health and Welfare Data Science Center, Ministry of Health and Welfare, Taiwan conducted the procedures of data linkage and scrambled personal identification. Based on their regulation, researchers can only take out summarized statistical results, without any raw data. In addition, the data were all analyzed anonymously. Therefore, we did not need to get consent from each patient.

### Study population

**Fig. 2:**
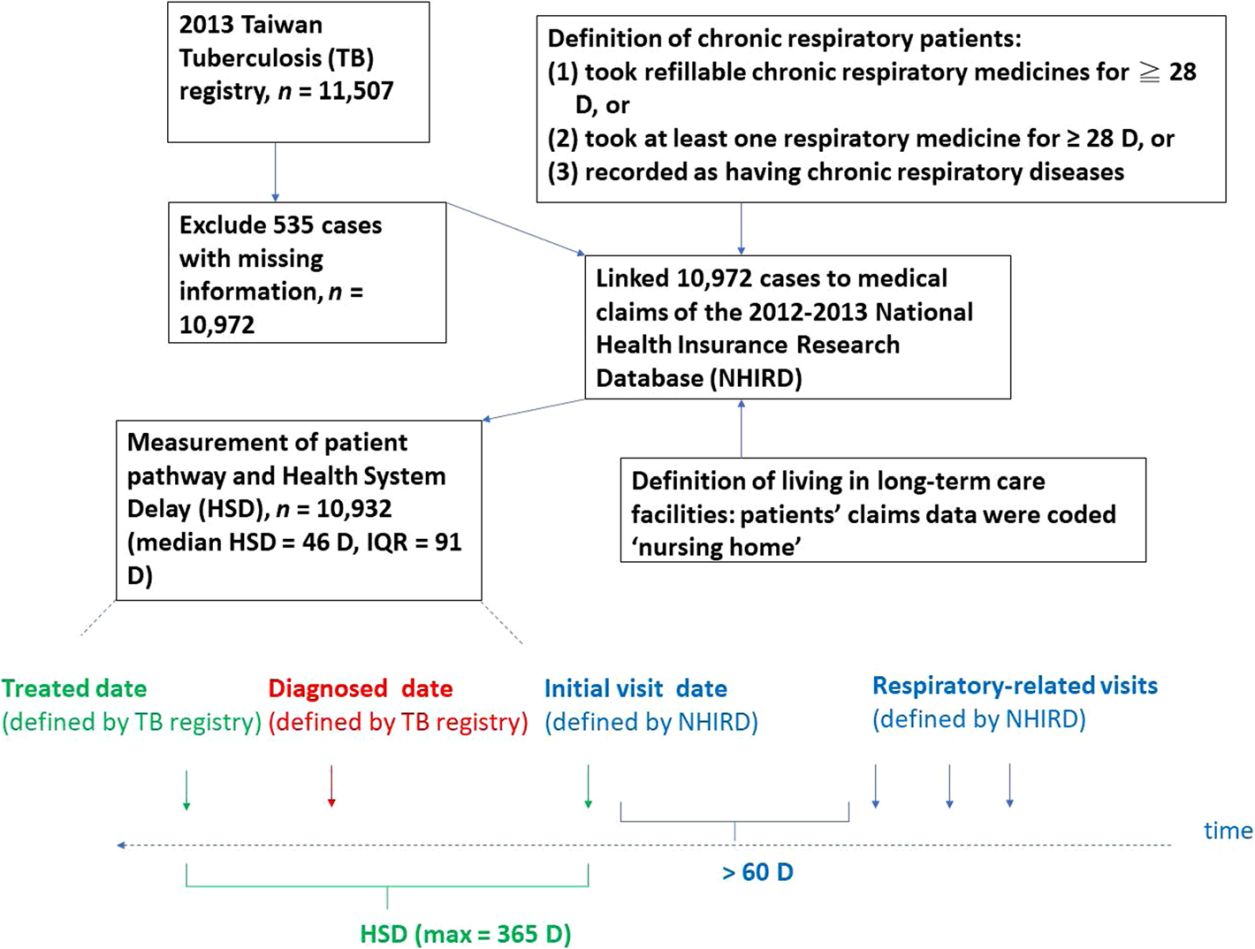
Flowchart of the study

The study population included all 11,507 patients with incident TB from the 2013 TB registry. Pulmonary TB diagnosis in Taiwan is based on clinical diagnosis, chest X-ray, sputum smear, and mycobacterium culture. The criteria of a confirmed case include a positive sputum smear/culture or clinical information, and the cases were approved by the government’s Center of Disease Control [17]. We excluded 535 patients due to important missing variables like results of sputum smear and culture. To analyze the care-seeking pathway of patients, we linked medical claim records from the 2012–2013 NHIRD to 10,932 Taiwanese in the TB registry (Fig. 2). NHIRD contains all the registration and claims data including utilization of inpatient and outpatient services for around 23,000,000 Taiwanese [18]. The coverage of NHIRD is more than 98% of the total population [19]. Under the coverage of universal health insurance, patients are free to seek health services from any clinician. A referral system is established, but tiered referral procedures are not mandatory [20]. Patients who prefer to directly visit tertiary care hospitals can do so without any referral, even for simple illnesses such as upper respiratory infection [20].

### Operational definitions

HSD is defined as the interval between the first medical consultation for respiratory-related diseases and the initiation of TB treatment (Fig. 2) [1]. To identify the first medical consultation of a TB patient from NHIRD, two sequential respiratory-related visits were treated as belonging to the same respiratory episode if the interval between the two visits was ≤60 days, and as different respiratory episodes if the interval was > 60 days [21]. The observation window for measuring HSD was 12 months before the start of TB treatment. Details on identifying the initial visit from NHIRD were described in our previous study [21]. Dates of TB diagnosis and treatment were defined by the 2013 TB registry. Chronic respiratory patients were patients who (1) took refillable chronic respiratory medicines for ≧28 days; or (2) took at least one respiratory medicine for ≧28 days; or (3) were recorded as having chronic respiratory diseases in NHIRD during the one-year observation window (Additional file 1: Table S1) [21]. Patients whose medical claims were coded ‘nursing home’ were recognized as living in long-term care facilities (Fig. 2).

### Health system delay and patient-pathway analyses

We treated HSD as time to event (treatment) data and investigated the HSD determinants by using a survival analysis. Cox proportional hazards regression was used to estimate the hazard ratios (HRs) of determinants in both univariable and multivariable models [22]. An HR < 1 indicated that the determinant was associated with a longer HSD and vice versa. We used SAS 9.3 (SAS Institute Inc., Cary, NC, USA) to perform the statistical analysis.

We further examined a patient’s care-seeking pathways from the first medical consultation to the initiation of TB treatment (Fig. 2). We marked the patient’s visits in chronological sequence and evaluated whether TB services (diagnosis and treatment) were provided on the date of initial visit. If a patient’s earliest date of TB diagnostic test (treatment) in the TB registry was on or before the initial visit date (treatment initiation date) in NHIRD, TB diagnosis (treatment) was provided. For those whose HSD was 0, TB treatment was provided at the initial visit.

## Results

In this cross-sectional study, incident cases from the 2013 TB registry were linked to the 2012–2013 NHIRD to conduct patient-pathway and HSD analyses (Fig. 2). Seventy percent of the total of 10,932 incident TB patients were male (Table 1). More than half of the patients (52.5%) were aged ≧65 years. Percentages of abnormal X-ray, initial sputum smear-positive, and initial culture-positive were 91.0, 30.0, and 63.3%, respectively.

**Table 1:** Characteristics of incident tuberculosis patients from the 2013 tuberculosis registry, Taiwan (*n* = 10,932)

Variable		*N* (%)	Median HSD^a^ (IQR^b^)	Median two-sample test	*p*-value
Gender	Male	7652 (70.0)	44 (89)	3.197	0.001
	Female	3280 (30.0)	49 (90)		
Age	<65	5223 (47.8)	33 (65)	−18.925	<0.001
	> = 65	5709 (52.5)	60 (121)		
Chest X-ray abnormal	Yes	9949 (91.0)	45 (90)	1.916	0.056
	No	983 (9.0)	49 (93)		
Sputum smear-positive (1st sample)	Yes	3279 (30.0)	34 (94)	−7.759	<0.001
	No	7653 (70.0)	49 (87)		
Sputum culture-positive (1st sample)	Yes	6919 (63.3)	45 (93)	0.424	0.671
	No	4013 (36.7)	46 (86)		
Chronic respiratory patient	Yes	4282 (39.2)	78 (160)	28.908	<0.001
	No	6650 (60.8)	32 (60)		
Long-term care facilities	Yes	420 (3.8)	140 (272)	12.202	<0.001
	No	10,512 (96.2)	44 (85)		
Health facility type of initial visit	Primary care clinic	4319 (39.5)	68 (110)	25.339	<0.001
	Hospital	6613 (60.5)	33 (71)		
District of initial visit	Eastern (reference)	429 (3.9)	41 (94)	9.006 (ChiSq)	0.109
	Taipei	3090 (28.3)	45 (84)		
	Northern	1301 (11.9)	42 (82)		
	Central	2029 (18.6)	45 (91)		
	Southern	1695 (15.5)	49 (101)		
	KaoPing	2388 (21.8)	47 (96)		

^a^*HSD:* health system delay

^b^*IQR:* interquartile range

For patients with chronic respiratory diseases (39.2% of 10,932), median HSD was significantly longer than for those without (78 days against 32 days). Patients living in long-term care facilities (3.8% of 10,932) had a dramatically longer delay (median HSD = 140 days) than others (median = 44 days). Compared to patients whose initial visits were at a hospital (median HSD = 33 days), patients whose initial visits were at a primary care clinic suffered from prolonged HSD (median HSD = 68 days). In terms of area, median HSD ranged from 41 to 49 days in six administrative districts of Taiwan. The overall HSD was 46 days (interquartile range = 91 days).

**Fig. 3:**
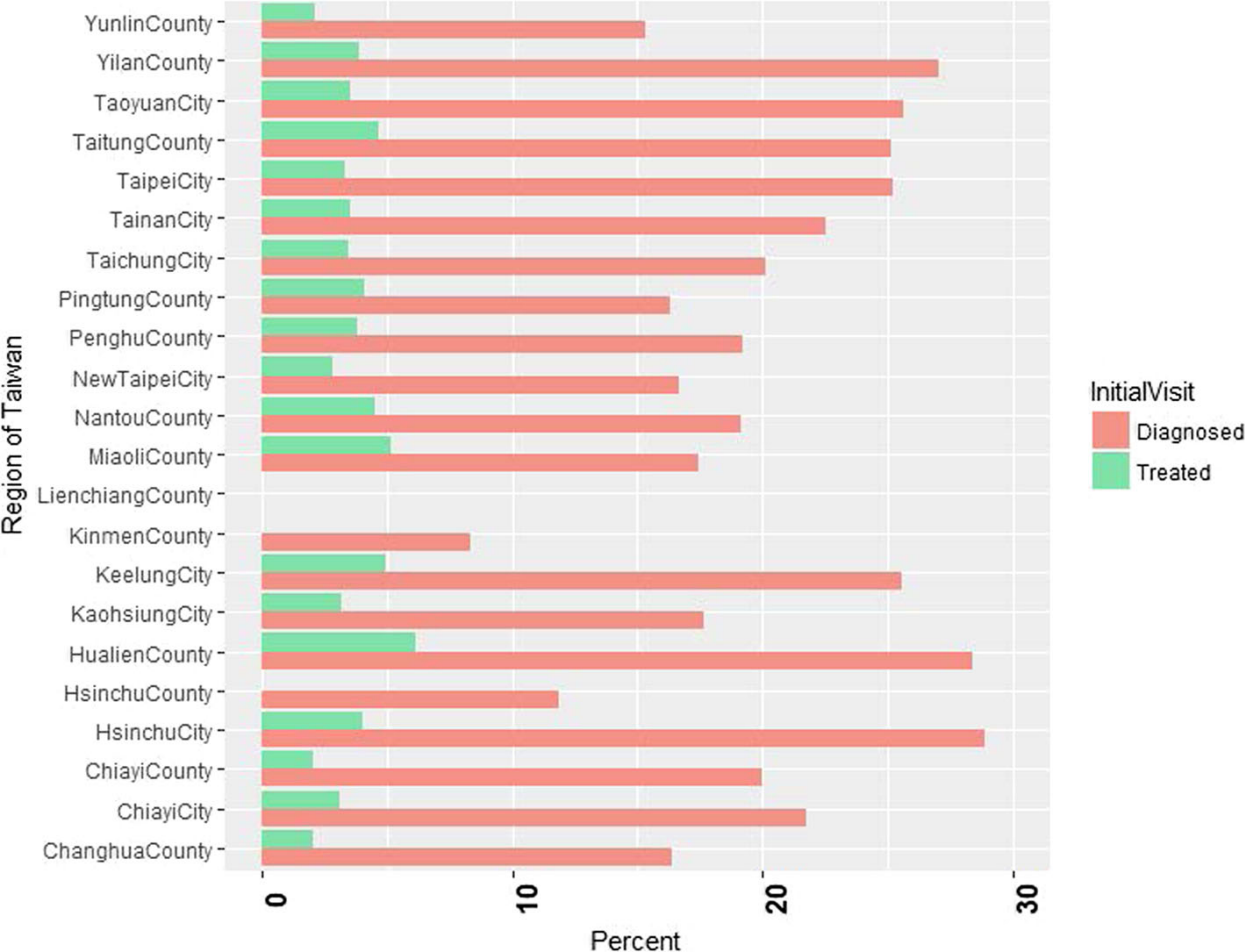
Percent of access to tuberculosis diagnostic and treatment services at the initial care seeking by region. Note that the percentages for access to tuberculosis diagnostic and treatment services at the initial visit are 0 in Lien-chiang County

We depicted the percentages of access to TB diagnostic and treatment services at the time of initial visit by 22 regions (cities and counties) (Fig. 3). Sixty percent of TB patients initiated their care visits at hospital (Table 1). Overall, the percentages of access to TB diagnostic and treatment services at the initial visit were 20.5% (2244/10,932) and 3.5% (380/10,932), respectively at the national level. In regions with more incident TB cases, lower percentages of patients received TB services at the initial visit. For example, 16.7% (*n* = 313) and 2.9% (*n* = 55) of patients received TB diagnosis and treatment at the initial visit in New Taipei City (total cases = 1887 in 2013) compared to 25.2% (*n* = 44) and 4.6% (*n* = 8) in Taitung County (total cases = 173 in 2013) [23].

**Fig. 4:**
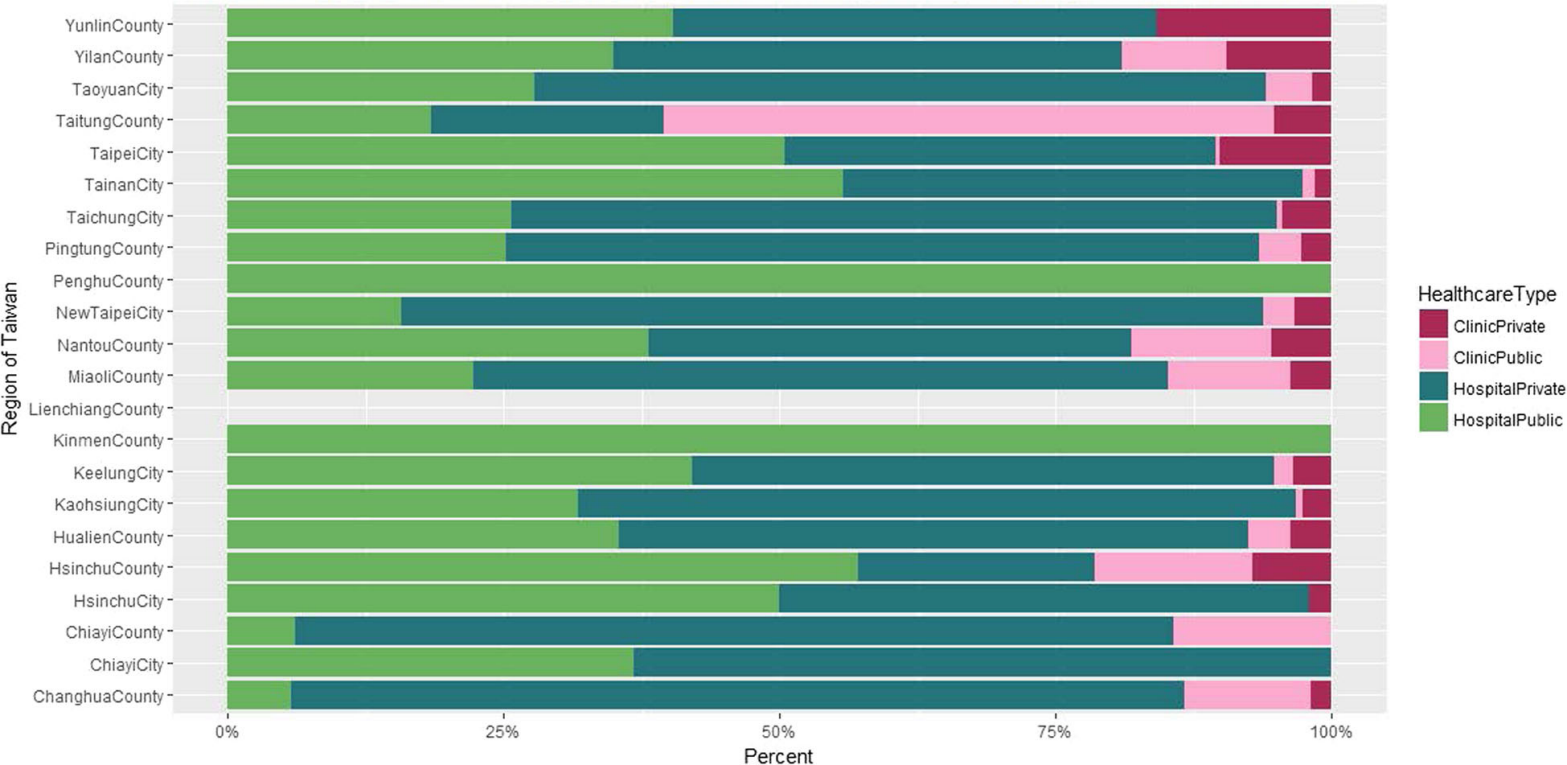
Percent of the initial visits where tuberculosis was diagnosed by hospital type and administration unit. Note that the percentage for access to tuberculosis diagnostic services at the initial visit is 0 in Lien-chiang County

**Fig. 5:**
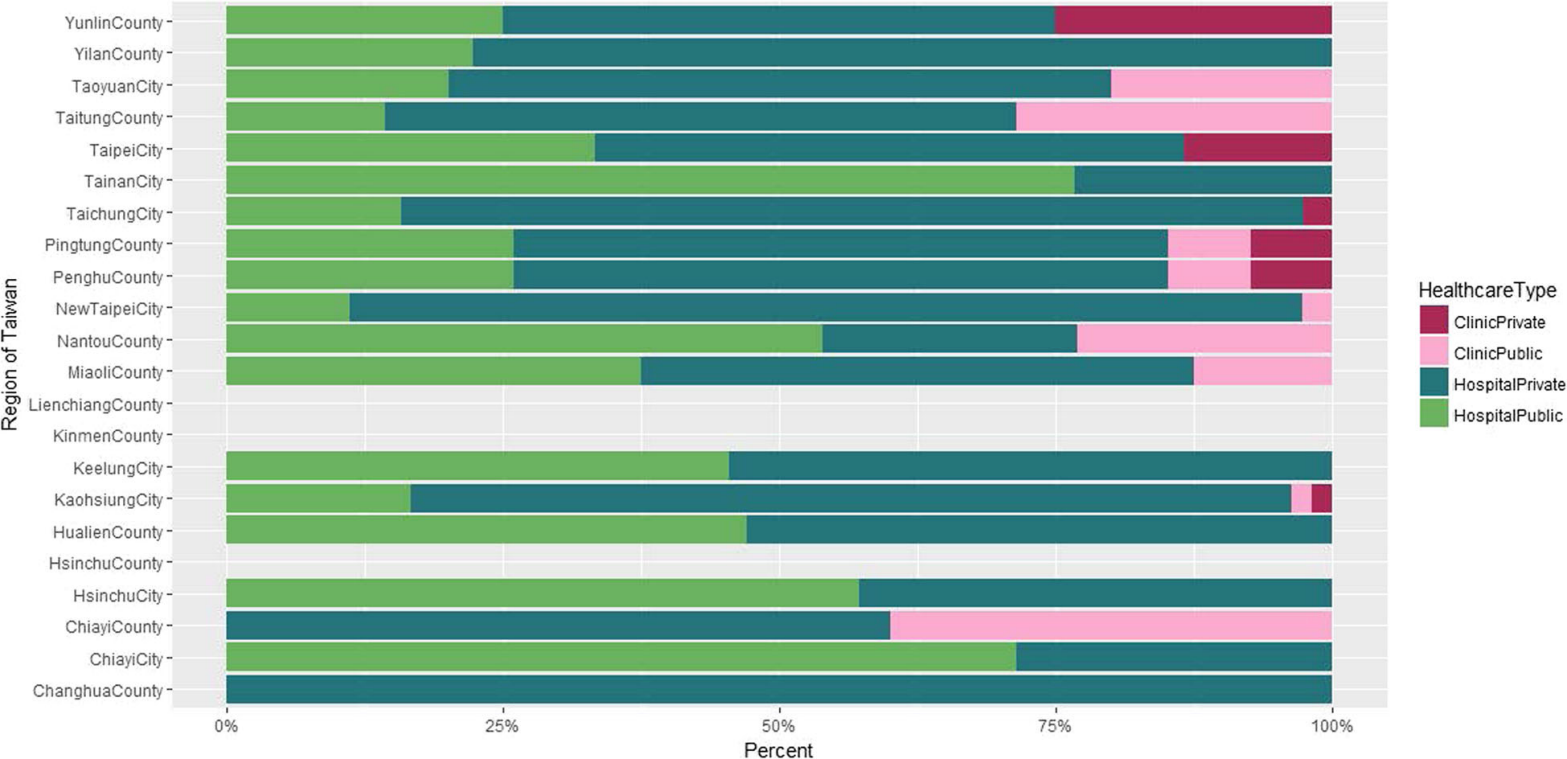
Percent of the initial visits where tuberculosis was treated by hospital type and administration unit. Note that the percentage for access to tuberculosis treatment services at the initial visit is 0 in Lien-chiang County

Among those receiving TB diagnosis (*n* = 2244) and treatment (*n* = 380) at the initial visit, we further examined what types of health facility (private clinic, private hospital, public clinic, and public hospital) they visited. We observed that hospitals (green colors) had a higher share than primary care clinics (red colors) across all regions except Taitung County (Fig. 4). Distributions for the percentage of TB treatment at the initial visit are similar to the pattern in Fig. 4, and even magnified (Fig. 5) where the share of private hospitals (dark green) becomes more dominant in a trend test (*p*-value< 0.001).

We portray the estimates of Cox proportional hazard model in Table 2 and Additional file 2: Figure S1. The median HSD differed by gender, age, severity, whether they also had chronic respiratory diseases, whether living in long-term care facilities, health facility type, and administrative district. In the multivariable survival analysis, patient’s risk factors for prolonged HSD included: female (adjusted HR = 0.921, 95% confidence interval [CI]: 0.884, 0.960) as compared to males; aged (≧65 years) (adjusted HR = 0.720, 95% CI: 0.692, 0.750) as compared to young patients (aged < 65 years old); non-severe (adjusted HR =0.721, 95% CI 0.683–0.760) as compared to severe (chest X-ray with cavities), and patients with chronic respiratory diseases (adjusted HR = 0.544, 95% CI: 0.522, 0.566). Health-system-related risk factors were: patients living in long-term care facilities (adjusted HR = 0.580, 95% CI: 0.525, 0.640); an initial visit to a primary care clinic (adjusted HR = 0.588, 95% CI: 0.565, 0.612) as compared to a hospital, and southern (adjusted HR = 0.887, 95% CI: 0.798, 0.987) as compared to eastern Taiwan.

**Table 2:** Cox proportional hazard model on health system delay (HSD) for incident tuberculosis patients in Taiwan, 2013 (*n* = 10,932)

Variable	Crude			Adjusted		
	Parameter (SD)	Hazard ratio (CI)	*p*-value	Parameter (SD)	Hazard ratio (CI)	*p*-value
Female (against male) (*n* = 3280)	−0.040 (0.020)	0.961 (0.922, 1.001)	0.053	−0.081 (0.021)	0.921 (0.884, 0.960)	<0.001
Age > =65 years (against age < 65 years) (*n* = 5709)	− 0.456 (0.019)	0.634 (0.610, 0.658)	<0.001	− 0.328 (0.020)	0.720 (0.692, 0.750)	<0.001
Non-severe (against chest X-ray with cavities) (*n* = 9297)	− 0.356 (0.026)	0.700 (0.664, 0.733)	<0.001	− 0.327 (0.027)	0.721 (0.683, 0.760)	<0.001
Patients with chronic respiratory diseases (*n* = 4282)	− 0.664 (0.020)	0.514 (0.494, 0.535)	<0.001	− 0.609 (0.020)	0.544 (0.522, 0.566)	<0.001
Long-term care facilities (*n* = 420)	− 0.615 (0.049)	0.540 (0.490, 0.596)	<0.001	−0.544 (0.050)	0.580 (0.525, 0.640)	<0.001
Primary care clinic (*n* = 4319)	−0.399 (0.019)	0.671 (0.646, 0.697)	<0.001	−0.530 (0.020)	0.588 (0.565, 0.612)	<0.001
District: Central (*n =* 2029)	−0.035 (0.053)	0.965 (0.869, 1.071)	0.501	−0.032 (0.053)	0.968 (0.872, 1.075)	0.544
District: KaoPing (*n =* 2388)	−0.061 (0.052)	0.940 (0.848, 1.042)	0.240	−0.090 (0.052)	0914 (0.824, 1.013)	0.086
District: Northern (*n =* 1301)	0.064 (0.055)	1.067 (0.956, 1.190)	0.246	−0.028 (0.055)	0.971 (0.871, 1.084)	0.604
District: Southern (*n =* 1695)	−0.091 (0.054)	0.913 (0.821, 1.015)	0.091	−0.119 (0.054)	0.887 (0.798, 0.987)	0.027
District: Taipei (*n =* 3090)	0.001 (0.051)	1.001 (0.905, 1.107)	0.987	−0.040 (0.051)	0.960 (0.868, 1.062)	0.429

## Discussion

Patient-pathway and HSD analyses were conducted to unveil the alignment between patient care seeking and the availability of prompt TB services among 10,932 incident cases in Taiwan. The results suggest that 20.5 and 3.5% of TB patients were diagnosed and treated at the initial visit in Taiwan. Risk factors related to prolonged HSD included female gender, aged > = 65 years, non-severe (chest X-ray without cavities), having chronic respiratory diseases, living in long-term care facilities, an initial visit to a primary care clinic, and southern areas of Taiwan.

Although approximately 60.5% of patients initiated care seeking at a hospital, HSD (median = 46 days) remains long compared with other countries (for example, median HSD = 15 days in Croatia) with an intermediate TB disease burden [24]. The low access to TB diagnosis (20.5%) and treatment (3.5%) at the initial visit indicates inefficiency in Taiwan’s health system. We also observed regional differences in access to TB services. For example, a relatively low percentage of prompt TB diagnosis and treatment at the initial visit and prolonged HSD in southern areas might require further examination. On the other hand, strengthening physician training at public hospitals and private clinics might improve the efficiency and timeliness of TB services in Taiwan [16].

Compared to our previous study [21], we further observed that patients with chronic respiratory diseases and those living in long-term care facilities have prolonged HSD. The non-specific nature of the symptoms of chronic respiratory diseases like cough is a risk factor associated with longer delays [25]. In addition, demographic changes have increased the number of elderly individuals for whom age-related immunosenescence may increase LTBI activation risk, especially in settings with vulnerable individuals [26]. A delayed diagnosis of TB in a long-term care facility may lead to nosocomial exposure [27]. Therefore, necessary resource allocation, like adoption of GeneXpert MTB/RIF in the health care system and testing by interferon-gamma release assay (IGRA) in long-term care facilities, might be considered [28].

HSD can be further divided into three interconnected components (Fig. 2): a) arousing suspicion time: the interval between the first medical consultation and the time when a TB diagnostic test is ordered; b) diagnosis time: the interval between ordering a TB diagnostic test and a positive result; c) treatment time: the interval between the positive diagnostic test and the initiation of TB treatment [29, 30]. According to the 2013 TB registry, we observed that the median delays for b) and c) were 5 and 0 days, respectively. Therefore, we estimated that the contribution of a) might be around 41 days (median) when matching patients’ medical claims in the NHIRD.

Unlike Hanson et al.’s approach [31] using either a TB prevalence survey or demographic and health surveys to conduct PPA, our estimates of TB diagnosis and treatment access at the initial visit were obtained from the national TB registry and individual medical claims (NHIRD). While a TB prevalence survey provides TB-specific care-seeking data, the sample size of patients confirmed to have TB is usually small and will not allow for robust subnational analysis [31]. On the other hand, indicators from population-based demographic and health surveys are not TB-specific [31]. Our estimates from a total of 10,932 observations are not prone to small sample size and capture care-seeking pathways for patients with respiratory-related symptoms.

However, this study still has several limitations. Firstly, because one-fourth (*n* = 2848, 26.1%) of the total observations from the 2013 TB registry lacked the treatment outcome information, we didn’t include treatment outcomes in our study, which is important for a comprehensive PPA. Secondly, we only conducted a one-year retrospective study. The estimate of HSD and the share of access to TB diagnosis and treatment at the initial visit might be underestimated. In addition, the identification of chronic respiratory patients might be misclassified due to a short observation window (12 months), and a validation of the operational definition (28-day cutoff) is warranted. Finally, since being diagnosed with TB is a dynamic process, the percentage of access to TB diagnostic (treatment) services might be underestimated by comparing the date of initial visit (treatment initiation) in NHIRD and the date of TB diagnostic test (TB treatment) in the TB registry, which could be improved by reviewing the TB-related procedures from medical claims of NHIRD [30].

## Conclusions

The low share of access to TB diagnostic and treatment services at the initial visit and the prolonged HSD indicate inefficiency in the health care system. Strengthening training of physicians at public hospitals and health workers at nursing homes might improve the efficiency and timeliness of TB diagnosis and treatment. In addition, we suggest that long-term care facilities and primary health care clinics might need to pay more attention on early identification of TB cases to avoid subsequent delays.

## Supplementary information

**Additional file 1: Table S1.** Drug lists associated with chronic respiratory medicines

**Additional file 2: Figure S1.** Kaplan Meir plots (direct adjusted) for health system delay by risk factors

## Data Availability

The raw data are confidential and cannot readily be shared. Researchers need to obtain permission from the Institutional Review Board of their institutions and apply for access to the data from the Health and Welfare Data Science Center, Ministry of Health and Welfare, Taiwan.

## Abbreviations

CI: Confidence interval
DOTS: Directly observed therapy, short-course
HRs: Hazard ratios
HSD: Health system delay
IGRA: Interferon-gamma release assay
IQR: Interquartile range
LTBI: Latent tuberculosis infection
NHIRD: National Health Insurance Research Database
PPA: Patient-pathway analysis
TB: Tuberculosis
