# Supplementary material for: Patient and health care system characteristics are associated with delayed treatment of tuberculosis cases in Taiwan
Source: BMC Health Serv Res. 2019 Nov 19;19:846. doi: 10.1186/s12913-019-4702-0 (PMC6862853; doi:10.1186/s12913-019-4702-0)
Supplement: Supplementary file 1 — Additional file 1: Table S1. Drug lists associated with chronic respiratory medicines [file 12913_2019_4702_MOESM1_ESM.docx]

**Additional file 1: Table S1.** Drug lists associated with chronic respiratory medicines

| Type | Medicine |
| --- | --- |
| Mucolytic | Acetylcysteine  Acetylcysteine Sodium  Ambroxol HCl  Compound Opium and Glycyrrhiza Mixture  Mesna  Cough Mixture |
| Antitussive agents | Codeine Phosphate  Dextromethorphan HBr |
| Bronchodilator | Albuterol Sulfate  Aminophylline  Procaterol HCl  Formoterol and Budesonide  Isoproterenol HCl  Pseudoephedrine HCl  Tiotropium |
| Asthma drugs | Budesonide  Fluticasone Propionate  Montelukast Sodium |
| Influenza drugs | Oseltamivir Phosphate |
