# Supplementary material for: Patient and health care system characteristics are associated with delayed treatment of tuberculosis cases in Taiwan
Source: BMC Health Serv Res. 2019 Nov 19;19:846. doi: 10.1186/s12913-019-4702-0 (PMC6862853; doi:10.1186/s12913-019-4702-0)
Supplement: Supplementary file 2 — Additional file 2: Figure S1. Kaplan Meir plots (direct adjusted) for health system delay by risk factors [file 12913_2019_4702_MOESM2_ESM.docx]

**Additional file 2: Figure S1.** Kaplan Meir plots (direct adjusted) for health system delay by risk factors

| a) Overall  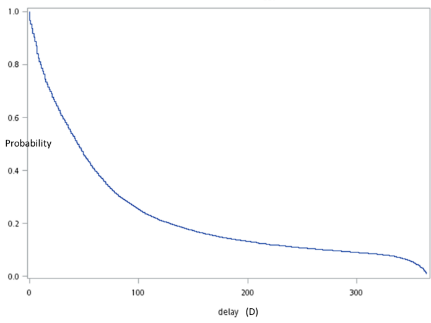 | b) Gender  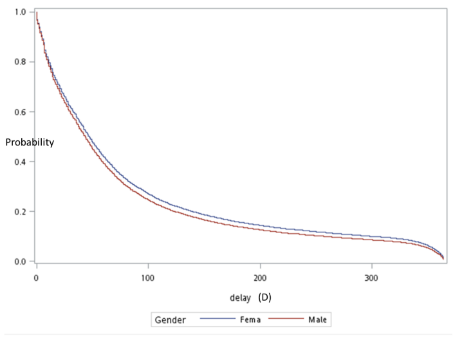 | c) Age  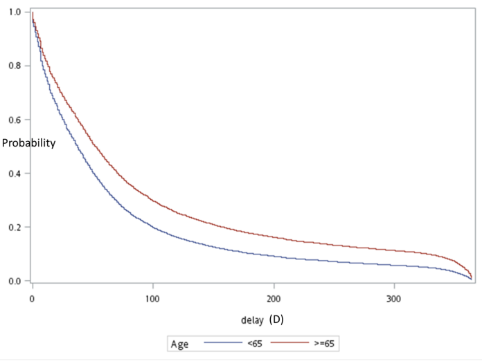 |
| --- | --- | --- |
| d) Non-severity  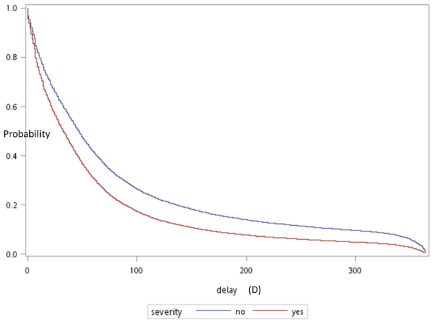 | e) With chronic respiratory diseases 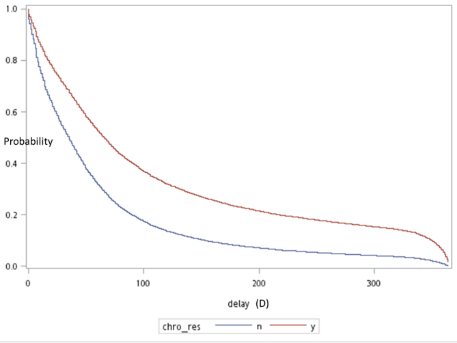 | f) Long-term care  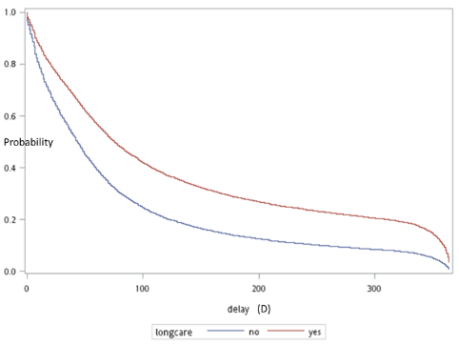 |
| g) District  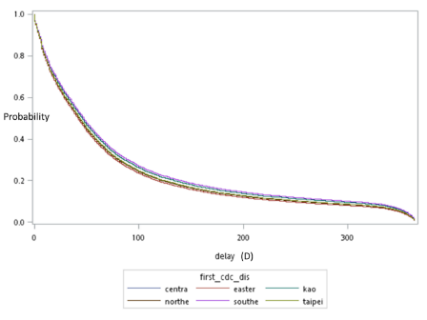 | h) Primary care clinic  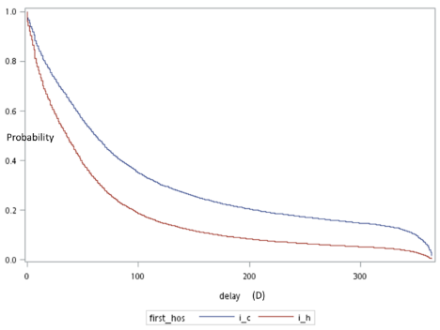 |  |
